# Supplementary material for: Collagen methionine sulfoxide and glucuronidine/LW-1 are markers of coronary artery disease in long-term survivors with type 1 diabetes. The Dialong study
Source: PLoS One. 2020 May 13;15(5):e0233174. doi: 10.1371/journal.pone.0233174 (PMC7219747; doi:10.1371/journal.pone.0233174)
Supplement: S1 Fig — (PDF) [file pone.0233174.s001.pdf]

Fig 1S

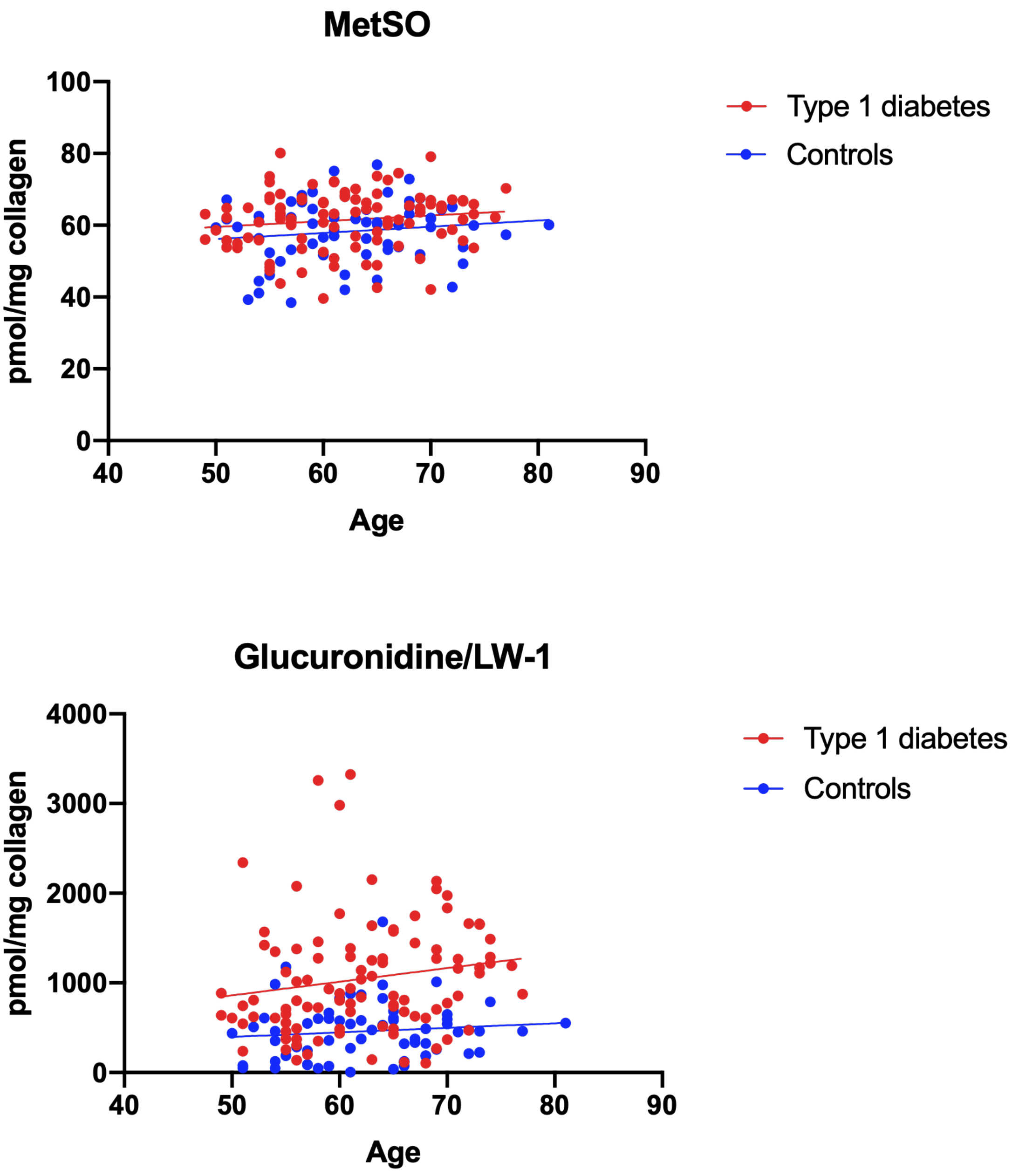
MetSO
pmol/mg collagen
100
80
60
40
20
0
40
50
60
70
80
90
Age
Type 1 diabetes
Controls
Glucuronidine/LW-1
pmol/mg collagen
4000
3000
2000
1000
0
40
50
60
70
80
90
Age
Type 1 diabetes
Controls

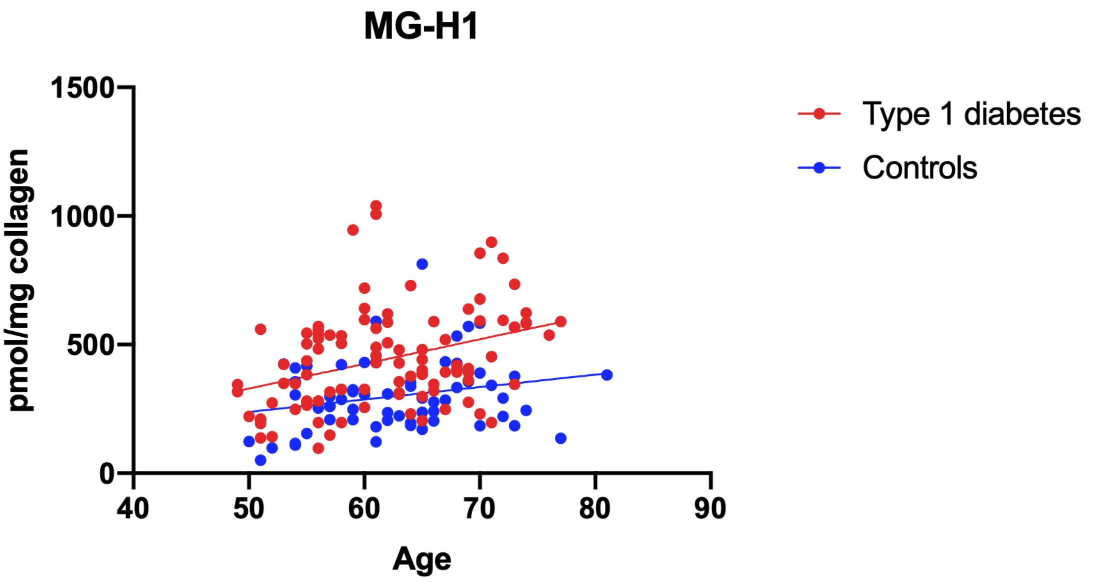
MG-H1
pmol/mg collagen
1500
1000
500
0
40
50
60
70
80
90
Age
Type 1 diabetes
Controls

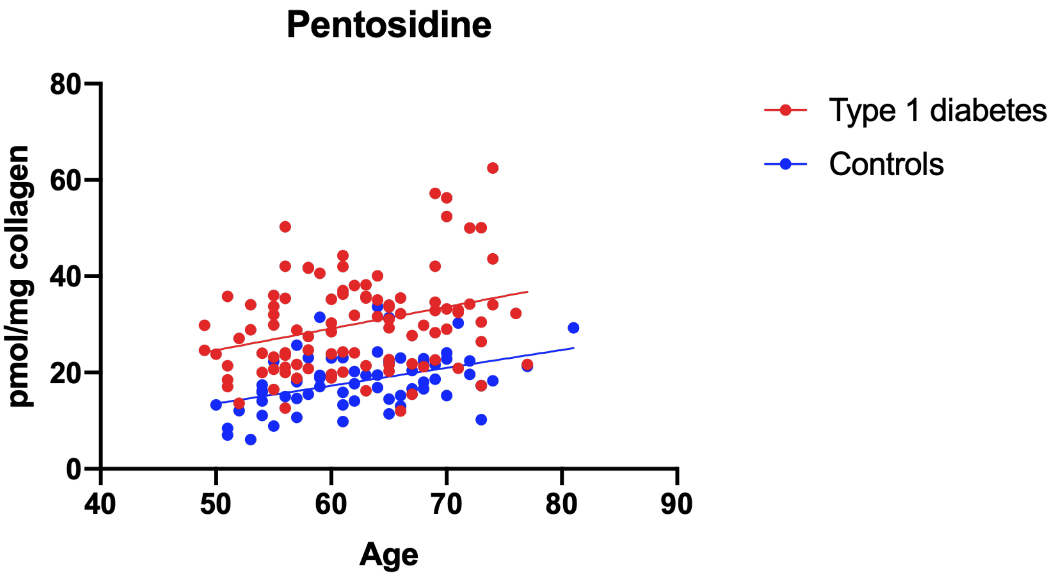
Pentosidine
pmol/mg collagen
80
60
40
20
0
40
50
60
70
80
90
Age
Type 1 diabetes
Controls

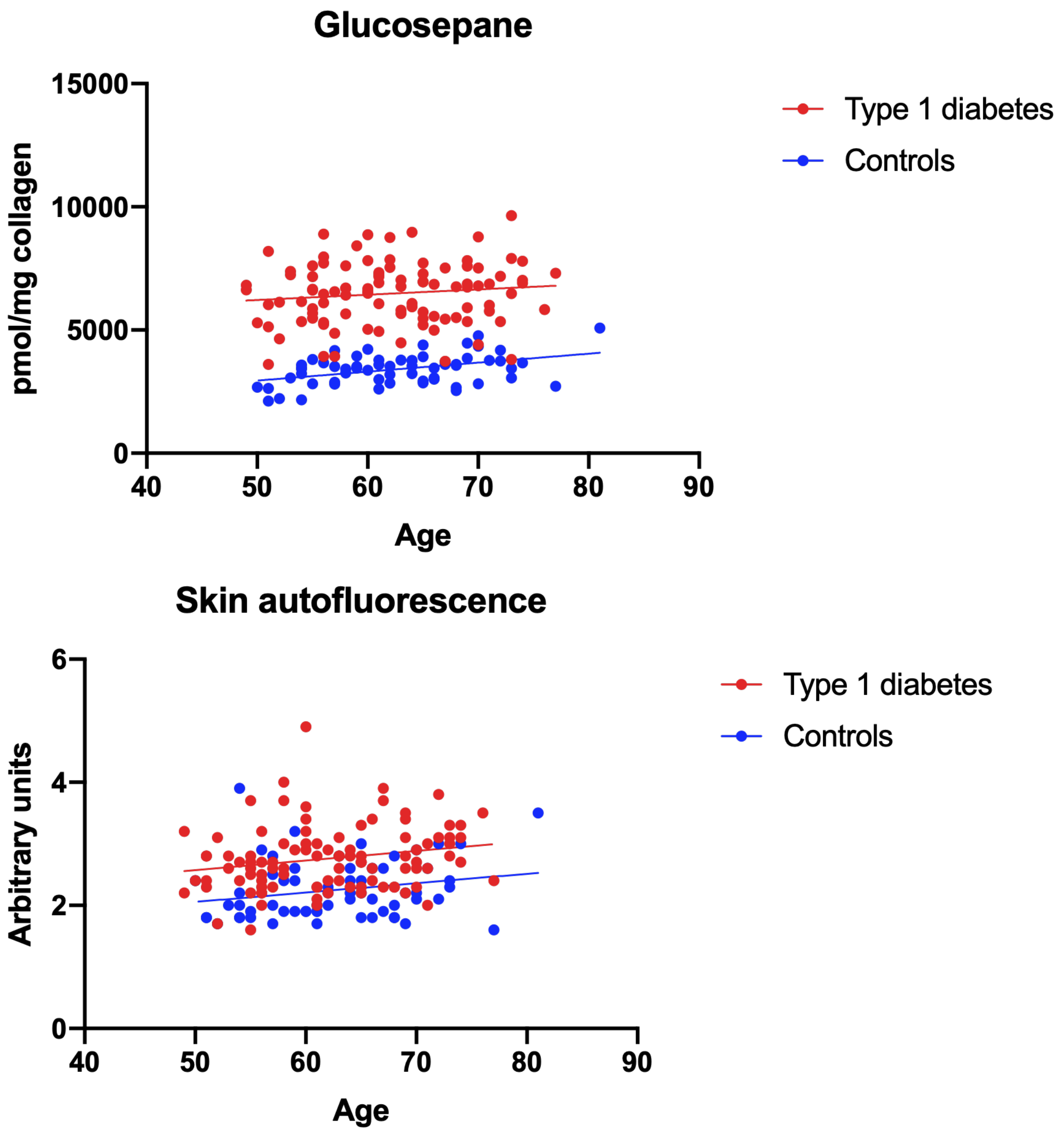

Fig 1S. Scatter plots with linear regression lines showing the age-related accumulation of methionine sulfoxide (MetSO), glucuronidine/LW-1, methylglyoxal hydroimidazolone (MG-H1), pentosidine, glucosepane and skin autofluorescence in the type 1 diabetes group in red and controls in blue.
