# Supplementary material for: Collagen methionine sulfoxide and glucuronidine/LW-1 are markers of coronary artery disease in long-term survivors with type 1 diabetes. The Dialong study
Source: PLoS One. 2020 May 13;15(5):e0233174. doi: 10.1371/journal.pone.0233174 (PMC7219747; doi:10.1371/journal.pone.0233174)
Supplement: S2 Fig — (PDF) [file pone.0233174.s002.pdf]

Fig 2S

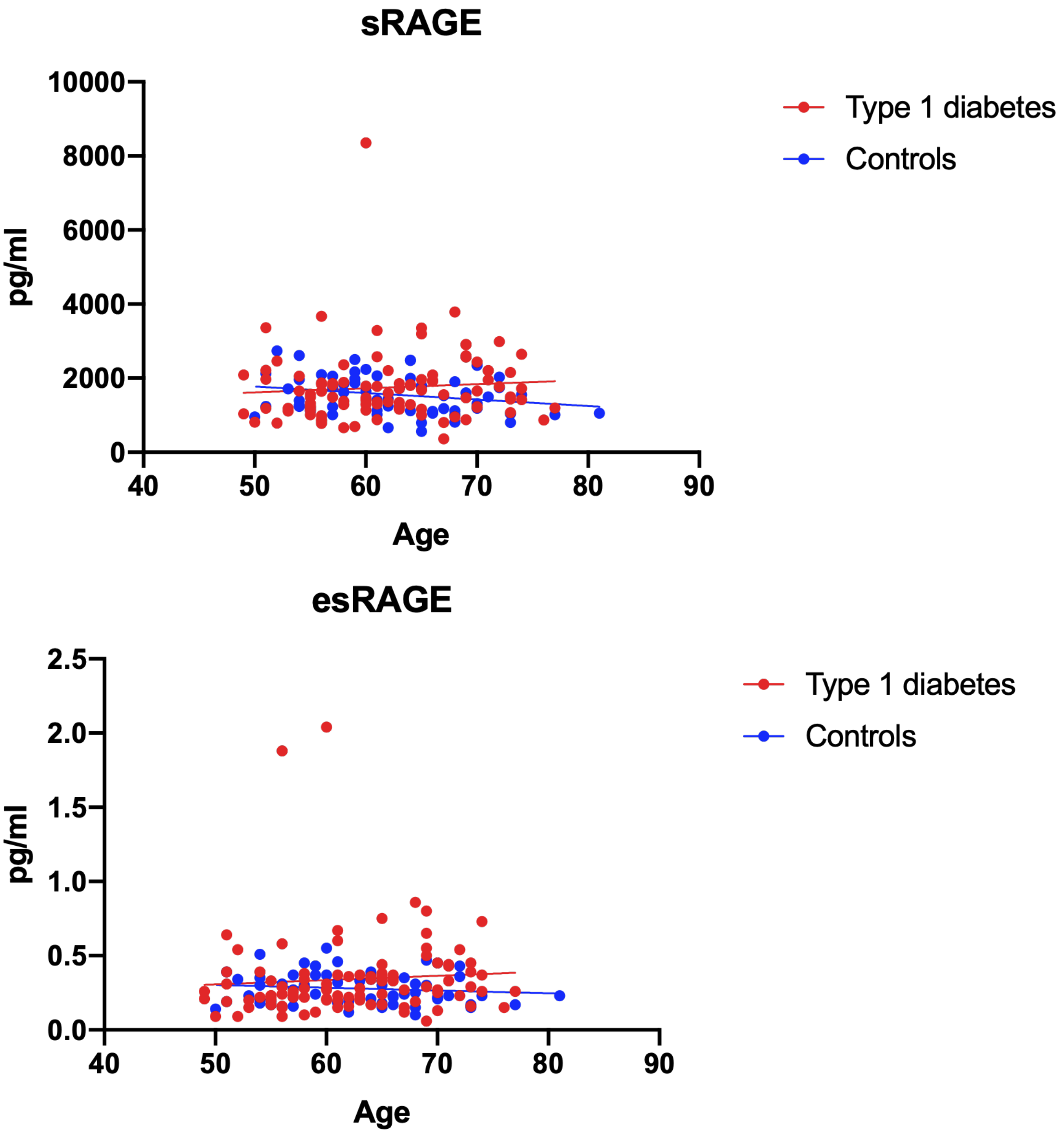

Fig 2S. Scatter plots with linear regression lines showing the age-related accumulation of sRAGE og esRAGE in the type 1 diabetes group in red and controls in blue.
