# Supplementary material for: Collagen methionine sulfoxide and glucuronidine/LW-1 are markers of coronary artery disease in long-term survivors with type 1 diabetes. The Dialong study
Source: PLoS One. 2020 May 13;15(5):e0233174. doi: 10.1371/journal.pone.0233174 (PMC7219747; doi:10.1371/journal.pone.0233174)
Supplement: Attachment — Submitted filename: Response to reviewers.docx [file pone.0233174.s004.docx]

We thank the editor for the thorough review of our manuscript and excellent comments. Please find our comments and changes to the manuscript below.

1. Provide fluorescence images of Fig 2.

Thank you for this suggestion. Figure 2 demonstrates the associations of a, MetSO, b, Glucuronidine/LW-1 c, MG-H1 and d, skin autofluorescence (measured using the AGE reader®) with categories of coronary artery disease in the diabetes group. MetSO, glucuronidine/LW-1 and MG-H1 were measured using LC-MS/MS. We agree that we could provide more raw data on the distribution of the levels of the variables, and we have now created scattergrams of the data versus age in both the diabetes and control groups as a supplement (Fig 1S).

2. Provide protein levels of RAGE.

Thank you for this suggestion. We measured sRAGE and esRAGE in serum using ELISA. They are expressed in pg/ml (Table 1). We are also providing the scattergram of these data in Fig 2S.

3. The discussion should be improved correlating these results with the literature.

We have added the following to the discussion: “*Previous studies have demonstrated that AGEs increase with age and duration (29), and indeed, the levels of AGEs in our study were higher than in previous studies with younger participants, exemplified by glucosepane (the most abundant AGE) which had a mean level of 6480 pmol ± 1254 in the diabetes group in our study compared to up to 4000 pmol/mg in patients with diabetes at age 30–40 years (17*).”

4. The manuscript is not linked to current conversations in the journal.

We have added the following to the discussion which refers to a study by den Dekker MA et al, PLoS One 2013: “*Skin autofluorescence have previously been suggested to be associated with both subclinical and clinical atherosclerosis independent of diabetes (35). While skin autofluorescence was associated with total plaque volume only in unadjusted analyses in the control group, it was associated with established CHD in the diabetes group, again signifying a role in advanced stages of the disease*.”
